# Introduction to the "Scoliosis" Journal Brace Technology Thematic Series: increasing existing knowledge and promoting future developments

**DOI:** 10.1186/1748-7161-5-2

**Published:** 2010-01-28

**Authors:** Stefano Negrini, Theodoros B Grivas

**Affiliations:** 1 ISICO (Italian Scientific Spine Institute), Via R Bellarmino 13/1, 20141 Milan, Italy; 2 Orthopaedic and Trauma Department, "Tzanio" General Hospital of Piraeus, Piraeus, Greece

## Abstract

Bracing is the main non-surgical intervention in the treatment of idiopathic scoliosis during growth, in hyperkyphosis (and Scheuermann disease) and occasionally for spondylolisthesis; it can be used in adult scoliosis, in the elderly when pathological curves lead to a forward leaning posture or in adults after traumatic injuries. Bracing can be defined as the application of external corrective forces to the trunk; rigid supports or elastic bands can be used and braces can be custom-made or prefabricated. The state of research in the field of conservative treatment is insufficient and while it can be stated that there is some evidence to support bracing, we must also acknowledge that today we do not have a common and generally accepted knowledge base, and that instead, individual expertise still prevails, giving rise to different schools of thought on brace construction and principles of correction. The only way to improve the knowledge and understanding of brace type and brace function is to establish a single and comprehensive source of information about bracing. This is what the ***Scoliosis*** Journal is going to do through the "Brace Technology" Thematic Series, where technical papers coming from the different schools will be published.

## Editorial

Bracing is the main non-surgical intervention in the treatment of progressive idiopathic scoliosis (IS) during growth, sometimes applied with a specific exercise program [1-8]. Bracing can also be used in adult scoliosis [9,10]. During growth, braces are also prescribed to patients with hyperkyphosis (HK) (and Scheuermanns disease) [11-17] and occasionally for spondylolisthesis [18-23]. And finally, they are applied in the elderly when pathological curves lead to a forward leaning posture [17,24,25] or in adults after traumatic injuries [26,27].

## Definition, goals of treatment and mechanisms of action

Bracing can be defined as the application of external corrective forces to the trunk with the following goals:

• during growth, to halt curve progression or to correct pathological spinal curves [28-31];

• in adulthood (mainly in the elderly), to avoid further collapse of already established pathological curves;

• after trauma, to allow proper vertebral healing and avoid progressive deformity [32,33].

To achieve these goals, rigid supports or elastic bands can be used [34,35] and braces can be custom-made or prefabricated [36-39].

Understanding the biomechanical action of a brace is of particular importance. The theoretical background for spinal correction is that the application of mechanical forces that reduce the pathological compression on given parts of the vertebral column while increasing it on others will result in a more symmetrical and natural loading that will, according to the literature:

• facilitate proper spinal growth [40-42],

• avoid progressive degeneration of the spine [40,43,44],

• unload damaged vertebral segments and allow proper remodelling [32,33].

Although this is an old concept, the theory has been reinforced over time and in the field of IS was recently summarized in the "vicious cycle" hypothesis [44], where it is proposed that lateral spinal curvature produces asymmetrical loading of the skeletally immature spine through movement and neuromuscular control, which in turn causes asymmetrical growth and hence progressive wedging deformity. In this respect, the role of the intervertebral disks in the progression of IS and in its possible correction using bracing has also recently been considered [45,46]. Conversely, bracing could establish a useful "virtuous cycle", and as a result could lead to gradual reduction of the asymmetry present in scoliosis [29,47]. In accordance with these theories, a novel concept describing a comprehensive model of IS progression, based on the patho-biomechanics of the deforming "three joint complex" was also recently presented [48]. All these theories are also relevant in treating HK and the traumatic spine with a brace.

An alternative hypothesis suggests that the use of braces leads to neuro-motor reorganization caused by the changes in external and proprioceptive inputs and movement resulting from the constraint of bracing [49-52]. According to this hypothesis, braces are considered the drivers of movement while they increase external and internal bodily sensations. This permanently changes motor behaviours, even when the brace is removed, and can have a long-term effect on bone formation. Also, this hypothesis can be easily applied at all pathologies and ages; this hypothesis can be considered true in terms of trunk behaviour and neuro-muscular organization, while its possible effect on growing bone needs further investigation.

Two other interesting and significant concepts to explain the actions of the brace have been discussed. One suggests that the brace provides mechanical support to the body (passive component), while the other suggests that the patient pulls his/her body away from pressure sites (active component) to correct the curve. Such divergent theories illustrate the complexity of this problem, but the most important point of brace treatment is to provide the three dimensional correction of the spinal deformity, and methodologies must be developed with this in mind [53].

## Expertise and actual Evidence Based Clinical Practice

The state of research in the field of conservative treatment is insufficient [54]. Interest in this specific topic decreased gradually from the 1970's to the 1990's, and only in the last decade has it improved, due to the efforts of the international scientific Society on Scoliosis Orthopedic and Rehabilitation Treatment (SOSORT) and its Journal (***Scoliosis***). What is clearly evident is that our understanding of brace effectiveness is still in its early stages of development. Braces are still named according to the town where it was developed [55-60] or the name of its inventor [47,61,62] or its theory [63-66]. No actual classification system exists to help distinguish one brace from another [65,67], and only very few comparisons among different braces have been published [68-70]. The only way to improve the knowledge and understanding of brace type and brace function is to establish a single and comprehensive source of information about bracing. This is what the ***Scoliosis*** Journal is going to do. A study of each brace type is clearly the first step toward an understanding of the common roots and the specific differences among them. This will, hopefully, stimulate even more research on bracing in the future. Let us start by documenting the knowledge of our brace experts, and then use this to derive useful commonalities and increase our general knowledge.

Brace effectiveness is questioned by some clinicians and researchers because there is not enough evidence published to support it [71-73]. A Cochrane Review on the topic in Adolescent IS has been published in the January, 2010 issue of the Cochrane Database [31]. It concludes that there is evidence in support of bracing, but much of it is of very low quality. There are even fewer published papers on HK brace treatment [15,16], and almost none exist in the treatment of spondylolisthesis [19]. While a few brace studies have been published in adults and in the elderly [25-27,32,33], a lot of research still needs to be done. Nevertheless, the existing results provide only weak evidence in favour of bracing in these clinical situations, and there is not a consensus that has been reached. Consequently, while it can be stated that there is evidence to support bracing, we must also acknowledge that today we do not have a common and generally accepted knowledge base, and that instead, individual expertise still prevails, giving rise to different schools of thought. In this respect, conservative experts have joined together in SOSORT, conceding that they may not share the same concept of the biomechanical action of corrective bracing on spinal curvature [29] even though they all agree on how to manage conservatively to obtain good results [74]. The first step must be to combine our collective knowledge and hold it up to scrutiny, so that a careful and thorough investigation of each theory can be completed.

We must also develop clear, consistent definitions of all the parameters used to measure brace effectivenes, because without this it will be impossible to compare the effectiveness of different brace types and the relative performance of the different centers involved. To help accomplish this, SOSORT is organizing its next consensus paper on this topic. It will be discussed at the 2010 Montreal SOSORT meeting and then published in ***Scoliosis***. This will be an important first step in evaluating the effectiveness of bracing. Recently, the standardization of criteria for Adolescent IS brace research has been established by the Scoliosis Research Society (SRS) Committee on Bracing and Non-operative Management [75,76]. The application of these criteria will greatly enhance research protocols exploring the effectiveness of bracing, and it is anticipated that much progress will be made in the near future as a consequence [31,77-80].

## Other issues in bracing

### The issue of compliance

In many studies, compliance with brace wear is measured by asking patients if they used their brace and how many hours of wear they had each day [81]. Some researchers added to this by looking for signs of wear on the brace to determine whether this matched with the patients report [82]. Some studies have reported that the amount of strap tension was highly correlated with the in-brace correction and the treatment outcomes [83]. Such variability in the way that compliance is measured has prompted the International Research Society on Spinal Deformities (IRSSD) to develop new tools to measure compliance more objectively. Recent improvement in electronics technology have given us new ways to accurately measure brace wear, and this is making research much more reliable. Some devices use temperature or humidity sensors for measuring purposes while others use force switches and pressure sensors [53].

### The issue of pressure being applied

How do we define optimal brace tightness? The in-brace correction depends on curve flexibility (which correlates highly with treatment outcomes) and the amount of pressure that the brace exerts. The optimal amount of pressure may be different in each patient. Most researchers have only recorded how much time the brace has been worn and do not record (or are unable to record) whether the brace has been worn correctly, especially in terms of the amount of pressure being applied. This is unfortunate because the absence of force measurement may result in a distorted view of the overall compliance [53].

### The issue of treatment time

Another important issue is the number of hours that a brace should be worn by the patient on a daily basis. Unfortunately, guidance given to the patient is generally based on 'clinical intuition'. The most commonly recommended treatment time is 23 hours per day. In recent years, the SRS has raised doubts as to whether part-time brace wearing is effective at all. If part-time treatment is effective then the question arises: How many hours per day is sufficient? There is no doubt that prediction of brace treatment outcome is difficult, but it becomes more so when treatment time is not standardized and we cannot accurately determine the risk of progression, the in-brace correction, or the compliance (both wear time and wear tightness) [53].

### The issue of bending radiographs

What is the best way to determine the flexibility of the spine? Although bending radiographs can provide accurate flexibility information, exposing growing children to additional radiation is undesirable [53]. Should radiographic data be used in scoliosis research on brace effectiveness and if yes, which way?

## Characteristics of articles published in the "Brace Technology" Thematic Series of the Journal

The articles published in the "Brace Technology" Thematic Series of the ***Scoliosis***Journal will comply with some essential characteristics and format. Manuscripts submitted for the series should be written on braces whose efficacy has been confirmed either in the very short term (at least six months), short term (end of growth) or long term (follow up beyond growth). All custom made or pre-fabricated corrective braces for spinal deformity (including non-idiopathic scoliosis, HK, Scheuermann's disease, spondylolisthesis, etc.) in all ages can be submitted and published in this section.

The "Brace Technology" articles describing specific braces should be organized as follows:

• **Introduction: **With general notes and goals of the study

• **History: **A short history of the brace

• **Theoretical principles**: How the brace might work (theory). General description of the mechanical principles of correction, the classification used for prescription, and the variations of the brace according to the curve pattern

• **The brace**: A description of the brace including photos of the front, back and sides in as many as possible curve types according to the classification used for prescription

• **Practical issues**. This should be divided in the following parts:

■**How to prescribe the brace: **principles of correction written in prescriptions by MDs

■**How to build the brace: **principles of construction by CPOs, with some photos; (see also the discussion regarding brace pressure above)

■**How to check the brace: **principles of checking by MDs and CPOs

■**Protocols: **description of the protocols generally used according to each clinical situations; (see criteria for bracing)

■**Everyday usage: **the number of hours per day that the patient will wear the brace

■**Exercises: **specific exercises while in the brace (if any), with photos of how to perform them

• **Results & case reports: **Short review of published results. The results should be divided into groups related to braces whose efficacy has been confirmed in the very short term (at least 6 months), short term (end of growth) or long term (follow-up beyond growth). Also, the type of study performed (case series, controlled) and the population considered must be reported. Moreover, 1 to 3 cases (of different curve types) fully documented with photos, clinical data and x-rays should be reported. However, patient consent must be obtained for this data and provided upon submission.

• **Discussion: **comparison with other braces based on the author's hypothesis; strengths and limitations, advantages and disadvantages

• **Conclusions**: with final remarks

• **The abstract **should be organized with the following sections: background, brace description and principles, results, conclusions

Moreover, in this thematic series, technical notes concerning particular details of brace construction could also be invited and published, to increase awareness and understanding of bracing. These articles can include theoretical comparison among braces, classification proposals, indications for future research directions, technical notes concerning particular details of brace construction, devices developed to accompany braces, and so on.

## Conclusion

***Scoliosis*** journal is focused on spinal deformity. Even though there is some early evidence in favour of bracing [31], the actual knowledge in the field does not yet allow us to classify the existing braces and categorize them beyond the names proposed by the original authors [65,67]. Consequently, the only possible way to increase our collective knowledge in the field is to publish what is being done today by clinicians with the most expertise in a systematic way, so to allow progressive comparisons and a deeper understanding. Moreover, discussion must be open among these experts, and these contributions will be accepted and published in this same thematic series of the journal. We are confident that with this new effort the journal will become an important source of information to the world of spinal deformity management, and will increase our understanding of how bracing effects the outcome of these problems. We do all this for the benefit of our patients.

## Competing interests

The authors declare that they have no competing interests.

## Authors' contributions

SN and TBG contributed equally in the manuscript drafting. The authors read and approved the final manuscript.
